# The Quest for Cellular Markers of HIV Reservoirs: Any Color You Like

**DOI:** 10.3389/fimmu.2019.02251

**Published:** 2019-09-20

**Authors:** Gilles Darcis, Ben Berkhout, Alexander O. Pasternak

**Affiliations:** ^1^Laboratory of Experimental Virology, Department of Medical Microbiology, Amsterdam UMC, University of Amsterdam, Amsterdam, Netherlands; ^2^Infectious Diseases Department, Liège University Hospital, Liège, Belgium

**Keywords:** HIV, biomarker, viral reservoirs, HIV latent reservoir, HIV persistence

## Abstract

Combination antiretroviral therapy (ART) suppresses human immunodeficiency virus (HIV) replication and improves immune function, but is unable to eradicate the virus. Therefore, development of an HIV cure has become one of the main priorities of the HIV research field. The main obstacle for an HIV cure is the formation of latent viral reservoirs, where the virus is able to “hide” despite decades of therapy, just to reignite active replication once therapy is stopped. Revealing HIV hiding places is thus central to HIV cure research, but the absence of markers of these reservoir cells greatly complicates the search for a cure. Identification of one or several marker(s) of latently infected cells would represent a significant step forward toward a better description of the cell types involved and improved understanding of HIV latency. Moreover, it could provide a “handle” for selective therapeutic targeting of the reservoirs. A number of cellular markers of HIV reservoir have recently been proposed, including immune checkpoint molecules, CD2, and CD30. CD32a is perhaps the most promising of HIV reservoir markers as it is reported to be associated with a very prominent enrichment in HIV DNA, although this finding has been challenged. In this review, we provide an update on the current knowledge about HIV reservoir markers. We specifically highlight studies that characterized markers of persistently infected cells in the lymphoid tissues.

## Introduction

Antiretroviral therapy (ART) allows the clinical management of the vast majority of human immunodeficiency virus (HIV) infected individuals, resulting in the prevention of AIDS and in the drastic reduction of the virus transmission risk. Unfortunately, ART is not curative. Interruption of therapy almost invariably leads to a quick viral rebound to pre-ART levels (1–6). Moreover, chronic immune activation and inflammation despite ART contribute to the excessive risk of non-AIDS events in ART-treated individuals (7). The etiology of persistent immune activation in the context of successful ART is considered multifactorial and involves microbial translocation, co-infections, metabolic disorders, regulatory T cell (Treg) deficiency, and decreased thymic function (8–11).

Presence of latent HIV reservoirs forms the major obstacle to an HIV cure. There is currently no consensus definition of the viral reservoirs due to their heterogeneous and dynamic nature. Viral reservoirs are commonly defined as cell types or anatomical sites that support the long-term persistence of replication-competent virus (12–14). This definition limits the HIV reservoirs to proviruses capable of triggering viral rebound after ART interruption (15). However, there is evidence that some defective proviruses that cannot reignite infection may still elicit immune activation through the production of viral proteins or novel antigens and thus participate in residual HIV pathogenesis (16–19). The eradication of this fraction of the HIV reservoir should therefore also be considered in the context of a durable cure. This concept led to extension of the HIV reservoir definition to all infected cells (20–22).

The definition of the HIV latent reservoir was initially restricted to transcriptionally silent proviruses (12). However, HIV post-integration latency is a multifactorial phenomenon controlled by mechanisms that operate not only at the transcriptional but also posttranscriptional levels (e.g., splicing and nuclear export of HIV RNA, translation, viral particle assembly, and maturation) (23, 24). These multiple blocks to virus production (25, 26) explain why subsets of latently infected cells in individuals on long-term ART can express HIV RNA and proteins (27–29). Such latently infected but transcription-competent cells have been defined as active HIV reservoirs (30–32). HIV latency has thus to be seen as a continuum with blocks to productive infection at different stages of the viral replication cycle in different populations of latently infected cells (33, 34). It has therefore been suggested to avoid the word “latent” when describing these cellular HIV reservoirs (22), and the term “leaky latency” has been proposed (Conference on Retroviruses and Opportunistic Infections 2015). However, it might be more practical to use a wider definition of HIV latency, integrating all the transcriptional and post-transcriptional blocks to virus production (34). Interestingly, those blocks are not “frozen” but can evolve over time. For instance, DNA methylation of the HIV promoter progressively increases during ART, possibly strengthening HIV latency (35). HIV reservoir expression also leads to clearance of infected cells due to immune surveillance or viral cytotoxicity, as suggested by a recent longitudinal HIV sequencing study (36). Prolonged ART is consequently associated with enrichment for proviral sequences that exhibit multiple features of deeper latency, such as positioning of the provirus in intergenic regions, in opposite orientation to host genes, and in either proximity to or increased distance from host transcription start sites and accessible chromatin (37).

In summary, a complex interplay of viral and host factors is continuously reshaping the HIV reservoirs. Moreover, this likely varies in different tissues, which are characterized by different anatomic properties and enriched for specific CD4^+^ T-cell subsets. It is therefore not surprising that mechanisms underlying HIV latency might differ between the peripheral blood and the tissues, as recently proposed (38). Viral and host markers of HIV reservoirs have been the focus of two recent reviews (39, 40). Here, we provide an update on some recent developments in the quest to distinguish the HIV reservoir cells from the plethora of cells in blood and tissues of an infected individual.

## Heterogeneous Nature of HIV Reservoirs

Understanding the cell types and anatomical sites that harbor latent HIV proviruses is essential for the design of therapeutic strategies to eradicate HIV from an infected individual. The developmental profile of a memory T cell has been increasingly well conceptualized, and is currently best characterized as a linear program during which highly immature, long-lasting memory T cells progressively transition toward more mature, differentiated and short-lived effector-memory cells (41, 42). HIV can persist in stem cell-like memory (T_SCM_), central (T_CM_), transitional (T_TM_), and effector (T_EM_) memory CD4^+^ T cells, in addition to naïve (T_N_) CD4^+^ T cells (43–47). Although the frequency of HIV-infected cells is lower in T_N_ compared to T_CM_, Zerbato et al. demonstrated that T_N_ harbor a large inducible reservoir of replication-competent HIV, suggesting that T_N_ cells may constitute an important HIV reservoir (48). Confirming these results, Venanzi Rullo et al. recently reported a large proportion of intact proviruses in T_N_ from two ART-treated subjects (49). Hiener et al. showed that, among memory CD4^+^ T cells, T_EM_ contain the larger proportion of intact HIV genomes (50). These studies indicate that the advent of improved culture- or PCR-based assays to measure the different reservoirs does seriously challenge the dogma that resting CD4^+^ memory T cells constitute the main HIV reservoir.

T cells can also be categorized according to functional properties, defined by specific antimicrobial properties or distinctive cytokine secretion patterns. Th1, Th2, Th17, Th9, regulatory T cell (Treg), and follicular T helper cell (Tfh) T-cell subpopulations have been identified based on functional polarization (42). Functionally polarized CD4^+^ T cells can support HIV persistence by distinct mechanisms related to their discrete functional profiles. Functional commitment toward a Th1 profile is possible during all stages of memory CD4^+^ T-cell development including the most immature and most durable subpopulations (51). Interestingly, proliferation of HIV-infected Th1 cells can play a crucial role in HIV persistence by maintaining the number of cells that harbor intact, replication-competent HIV (52). Recent evidence also highlights the contribution of long-lived Th17 cells to HIV persistence during ART, particularly in the gut (53). Th1/17 cells have been understood as a long-lasting subpopulation that may be somewhat more mature than Th17 cells, but retain the developmental program of long-lasting precursor cells for effector lymphocytes (54). CD4^+^ T cells enriched for Th1/17 polarization harbor high levels of HIV DNA in ART treated individuals and contribute disproportionately to the viral reservoirs that remain stable over many years of ART (45, 55).

Besides the gut and the lymph nodes (LN) that constitute major anatomical sites for HIV persistence, multiple other tissues and organs harbor HIV provirus in ART-suppressed individuals (56). Besides T cells, other cell types such as monocytes, astrocytes and tissue resident macrophages (splenic, alveolar, and microglia) may potentially serve as stable viral reservoirs in the tissues (20, 57–63). Given the difficulties in accessing tissue biopsies from humans, animal models such as humanized mouse models or SIV-infected non-human primate (NHP) models have been very helpful for examining HIV dynamics. These models have been used to show the persistence of HIV reservoirs in various tissues of HIV- or SIV-suppressed animals, with new tools allowing for demonstrating the intactness of these reservoirs (64–66).

Altogether, these studies highlight the highly heterogeneous nature of HIV reservoirs and underline the urgent need for identification of marker(s) of latently infected cells. Such markers could provide a “handle” to further dissect the complex and dynamic nature of the reservoirs and might even enable their selective targeting for eradication (67, 68). So far, no cellular marker has been discovered that is capable of identifying all HIV reservoirs. However, several HIV reservoir markers have recently been proposed and their further characterization could represent a significant step toward a better understanding of the HIV reservoirs.

## Immune Checkpoint Molecules

In chronic viral infections, high antigenic loads constantly stimulate T cells, resulting in progressive loss of function termed T-cell exhaustion (69, 70). During this period, T cells demonstrate increased expression of some inhibitory receptors, known as immune checkpoint molecules (ICs), which increase the threshold for activation, leading to suppressed immune responses. Such ICs include programmed cell death-1 (PD-1), cytotoxic T-lymphocyte-associated protein 4 (CTLA-4), lymphocyte activation gene 3 (LAG-3), T cell immunoglobulin, and ITIM domain (TIGIT), T cell immunoglobulin and mucin 3 (TIM-3), CD160, and 2B4 (CD244). Fromentin et al. showed that expression of PD-1, LAG-3, and TIGIT in CD4^+^ T cells was positively associated with the frequency of cells harboring integrated HIV DNA (71). More importantly, they observed that cells expressing these markers were enriched for HIV infection in several memory CD4^+^ T-cell subsets during ART. The majority of inducible HIV proviruses were found in memory CD4^+^ T cells that expressed at least one of these markers (71). More recently, the same group demonstrated a modest enrichment for HIV capsid (CA-p24)-producing cells (5.4-fold) in ART-suppressed individuals-derived cells displaying a CD45RA^−^, α4β1^+^, TIGIT^+^ phenotype (72). In line with these studies, Fromentin et al. recently reported that *ex vivo* HIV reactivation from latency is enhanced by a PD-1 blockade in CD4^+^ T cells from ART-suppressed individuals (73), although another group could not demonstrate such effect (74). In addition, Hurst et al. found that the expression of ICs measured prior to ART predicts time to viral rebound following ART interruption, confirming that T-cell exhaustion markers can identify latently infected cells with a higher tendency to viral transcription (75).

LN PD-1^+^/Tfh cells, as well as their circulating counterpart in peripheral blood (CXCR3^+^CD4^+^), represent a major cellular location for persistent virus in untreated and treated HIV-infected individuals, as demonstrated by the Perreau group (76–78). Notably, Tfh are localized to the germinal centers within the LN B-cell follicles, which are an immunologically privileged site with restricted CTL function (79). This may explain why PD-1^+^/Tfh cells are enriched in replication-competent HIV as compared to any other PD-1 negative memory CD4^+^ T-cell population isolated from blood or LN (77). However, intracellular concentrations of a number of antiretroviral drugs have been shown to be reduced in LN compared to peripheral blood (80–82), providing another possible reason for the persistence of infectious virus in the LNs. Interestingly, McGary et al. reported that CTLA-4^+^PD-1^−^ memory CD4^+^ T cells significantly contribute to SIV persistence in ART-treated rhesus macaques (83). CTLA-4^+^PD-1^−^ memory CD4^+^ T cells, a subset comprised predominantly of Tregs, were shown to be enriched for SIV DNA in several tissues and to contain replication-competent and infectious virus. In contrast to PD-1^+^/Tfh cells, SIV-enriched CTLA-4^+^PD-1^−^ Treg cells localized outside the LN B-cell follicle. Moreover, in HIV-infected individuals on ART, CTLA-4^+^PD-1^−^ memory CD4^+^ T cells harboring HIV DNA also persisted outside the B-cell follicle and contributed to the long-term latent viral reservoir (83).

Better definition of the parameters that dictate exhaustion is promoting the development of methods that prevent functionally inferior responses or “revitalize” them (84). IC inhibitors are already currently used to treat malignancies. This treatment would work for HIV infection in two ways: through boosting HIV-specific CD8^+^ T-cell effector function and through reactivating HIV expression from latency (85, 86). However, the case reports and clinical trials investigating the use of IC blockers have produced conflicting results (87, 88). Toxicity also appears as an important concern (89).

## CD2, CD30, and CD20

Using an *in vitro* primary CD4^+^ T-cell model for HIV post-integration latency (90), Iglesias-Ussel et al. studied the expression profile of latently infected CD4^+^ T cells (91). Among the markers identified in this study, CD2 was selected for further analysis. Resting memory CD4^+^ CD2^high^ T cells harbored higher HIV DNA copy numbers compared with the other cell subsets, although the fold enrichment was limited (5.7-fold). Iglesias-Ussel et al. further showed that CD4^+^ CD2^high^ T cells could be stimulated to express high levels of HIV RNA, although no evidence was provided that infectious viruses could be produced (91). In contrast to the other markers described in this review and for which some tissue data is available, the relevance of CD2 still has to be confirmed in lymphoid tissues.

CD30 is expressed in Hodgkin and other aggressive lymphomas and on a small fraction of lymphocytes in healthy individuals (92). CD30 expression can be triggered by infections with several viruses, including human T-cell lymphotropic virus and Epstein-Barr virus (92). It is known for more than 20 years that triggering CD30 may play an important role in both HIV replication and HIV-infected CD4^+^ T-cell death (93). More recently, Hogan et al. demonstrated that CD30^+^CD4^+^ T cells were significantly enriched for cell-associated HIV RNA but not for HIV DNA in several individuals regardless of ART use (94). CD30 expression and HIV transcriptional activity co-localized in gut-associated lymphoid tissue from ART-treated or ART-naïve individuals. Following *ex vivo* culture of peripheral blood cells in the presence of brentuximab vedotin, an anti-CD30 antibody-drug conjugate, they observed a significant reduction in total HIV DNA. These data suggested that CD30 may be a relevant HIV therapeutic target as it is not expressed on a vast majority of cells (93). Subsequently, the same group studied the impact of 3 doses of brentuximab vedotin therapy for Hodgkin lymphoma on HIV persistence and immune phenotype in an individual on long-term ART (95). An encouraging but transient reduction of CD4^+^ T cells expressing CD30 was observed, accompanied by reductions in CD4^+^ T-cell–associated HIV RNA, low-level plasma viremia, and to lesser extent HIV DNA levels (95). The reason for the lack of a sustained effect of brentuximab vedotin on CD30-expressing T cells is currently unknown.

Very recently, Serra-Peinado et al. reported a higher percentage of HIV RNA^+^ cells among cells expressing CD20 (96). CD20 is a known B-cell surface marker but is dimly expressed on a small subpopulation of CD4^+^ T cells. Although the contribution of CD20^dim^CD4^+^ T cells to the total pool of HIV RNA^+^ cells was modest (median of 18.6% in ART-treated and 25.0% in viremic HIV-infected individuals), *ex vivo* treatment of primary peripheral blood mononuclear cells (PBMCs) from ART-suppressed individuals with the anti-CD20 monoclonal antibody Rituximab appeared to reduce the pool of HIV RNA^+^ cells when combined with latency-reversing agents (96). Further studies are needed to elucidate whether Rituximab treatment is also capable of reducing the total or intact HIV DNA reservoir.

## CD32a

CD32a, also known as Fc gamma receptor IIa (FcγIIa), is the low-affinity receptor for the immunoglobulin G Fc fragment that is highly expressed on myeloid cells and expressed on a small subset of T cells (97, 98). CD32a was recently proposed by Descours et al. as a marker of HIV reservoir cells (99). Notably, CD32 has already been identified by an earlier study among several surface markers overexpressed in latently infected vs. uninfected CD4^+^ T cells in a primary CD4^+^ T-cell model for HIV latency (91). In contrast to all the markers described above that are associated with very limited HIV DNA enrichment, an extremely high (~1,000-fold) enrichment in HIV DNA was observed by Descours et al. in CD4^+^ T cells with high CD32a expression as compared to CD32^−^CD4^+^ T cells (99). They also demonstrated an enrichment for replication-competent proviruses in these cells, although not to a higher degree than the enrichment for total HIV DNA. If confirmed, these findings would represent a milestone in the efforts to develop a cure for HIV infection. However, a number of subsequent reports questioned whether CD32a would be a *bona fide* marker of the HIV latent reservoir (100–105), which decreased the initial enthusiasm. Some studies observed limited enrichment for HIV DNA in CD32^+^ cells in some participants but not in others (101, 102). Other reports could not demonstrate any enrichment for total HIV DNA or for replication-competent proviruses in CD32^+^ cells (100, 103–105).

**Figure 1:**
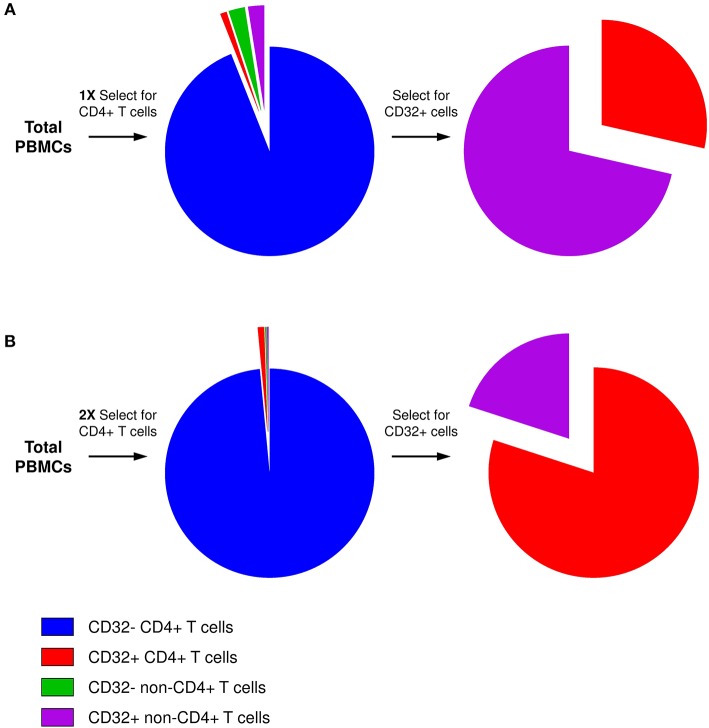
Presence of residual non-CD4^+^ T cells in the CD32^+^ cell fraction. **(A)** After one round of selection for CD4^+^ T cells, although the residual non-CD4^+^ T-cell contamination is low in general, the percentage of CD32^+^ cells among non-T cells is much higher than among CD4^+^ T cells. Therefore, after selection for CD32-expressing cells, the majority of CD32^+^ cells will be non-T cells. **(B)** Two consecutive rounds of CD4^+^ T-cell selection result in much purer CD4^+^ T-cell population, and the majority of CD4^+^ T cells is maintained even in the CD32^+^ fraction.

The key for this controversy likely resides in the technical difficulty to obtain a sufficiently pure population of *bona fide* CD32^+^CD4^+^ T cells. The frequency of CD32^+^ cells among antigen-presenting cells (APCs) is much higher than among CD4^+^ T cells. Therefore, even if the residual APC contamination of CD4^+^ T cells is low in general, APCs will be disproportionally overrepresented in the CD32^+^ fraction, as reported by several groups (101, 103) and also observed in our initial attempts to purify CD32^+^CD4^+^ cells (Figure 1A) (106). The excess of residual non-T cells in the sorted CD32^+^ fraction can easily obscure the enrichment for HIV DNA in CD32^+^CD4^+^ cells. Alternatively, certain cell sorting strategies and/or settings might result in the isolation of T-B cell doublets or conjugates instead of *bona fide* CD32^+^CD4^+^ T cells, as reported by two groups (105, 107). Because another CD32 isoform, CD32b, is highly expressed on the surface of B cells, and consequently on T-B cell doublets or conjugates, the latter cells may be preferentially recognized during FACS sorting for CD32^+^CD4^+^ cells as most anti-CD32 antibodies do not discriminate between the CD32a and CD32b isoforms. The extracellular domains of these two proteins are very similar, and no CD32a-specific antibody is available yet. Depending on the sorting settings and/or antibody titers, *bona fide* CD32^+^CD4^+^ T cells, which likely express less CD32 molecules per cell than T-B cell doublets/conjugates, might be missed, unless specific efforts are made to thoroughly deplete the CD32^+^ non-T cells.

To overcome these problems, we performed two consecutive rounds of CD4^+^ T-cell negative selection by magnetic cell sorting before we started with CD32^+^ cell isolation. This sequential cell purification strategy could efficiently deplete the vast majority of contaminating non-T cells, including any T-B cell doublets, which allowed us to obtain a purified population of CD32^+^CD4^+^ cells (Figure 1B) (106). Remarkably, we demonstrated a progressive increase in HIV DNA enrichment in CD32^+^CD4^+^ cells with further purification of these cells, and in the purified population we measured a very prominent enrichment for HIV DNA (average, 292-fold) (106), confirming the results of Descours et al. (99). Interestingly, several groups found that the majority of peripheral blood CD32^+^CD4^+^ T cells express the activation marker HLA-DR (100–102, 106, 108, 109). Although it has been shown previously that HIV can establish latent infection in activated CD4^+^ T cells (110, 111) and that the peripheral blood HIV DNA load is higher in activated compared to resting cells in ART-treated individuals (112), the existence of latently infected CD4+ cells that are activated, and therefore relatively short-lived, suggests continuous replenishment of this component of the reservoir by cellular proliferation (113). At present, however, high HLA-DR expression on CD32^+^CD4^+^ cells should be interpreted with caution, as it cannot be ruled out that the HLA-DR signal may partly originate from the non-T cells within the contaminating CD32^+^ T-non-T cell doublets or conjugates, as recently demonstrated by Thornhill et al. (107).

Persistence of CD32^+^ cells has also been studied in tissues. Abdel-Mohsen et al. showed that a high percentage of HIV-expressing cells localized within the B-cell follicle co-express CD32a, whereas CD32a single-positive cells were rarely observed (100). These RNA *in situ* hybridization (ISH) experiments showing the co-expression of CD32a RNA and HIV RNA in lymphoid tissue support the notion that CD32a is associated with transcriptionally active tissue reservoirs. Similar results were recently obtained by Vásquez et al. in the gut tissue (114). By using ISH-based methods, they demonstrated co-expression of HIV and CD32a mRNA, with most of the CD3^+^CD32^+^ cells in the gut co-expressing HIV RNA. Moreover, Thornhill et al. recently found that the T cells within the CD32^+^ T-B cell doublets in the gut and tonsil tissues of ART-treated individuals were associated with a Tfh phenotype (107). As Tfh cells have been previously shown to harbor high levels of HIV RNA and infectious virus in LN (77), this may provide a clue why CD32^+^ cells are associated with HIV transcription in tissues.

Two groups studied co-expression of CD32a and other HIV reservoir markers in tissues. Noto et al. analyzed HIV transcription in LN memory CD4^+^ T-cell populations sorted for CD32a and PD-1 expression (108). The CD32^+^ and PD-1^+^ CD4^+^ T-cell populations overlapped to a large extent and CD32^+^PD-1^+^ cells were significantly enriched for HIV RNA compared to CD32^−^PD-1^−^, CD32^+^PD-1^−^, and CD32^−^PD-1^+^ cells. In the LNs, CD32^+^ and PD-1^+^ CD4^+^ T cells expressed high levels of the HIV co-receptors CCR5 and CXCR4, potentially making these cells a preferential target for HIV infection, as well as higher levels of activation markers, which can explain why these cells are characterized by a high level of HIV transcription (108). Hogan et al. sorted rectal biopsy cells for the expression of CD32a and CD30 (94). The highest HIV RNA and DNA levels from rectal tissue-derived cells were measured in cells co-expressing CD30 and CD32. These two studies demonstrate that different HIV reservoir markers can be co-expressed on CD4^+^ T cells, raising the possibility that there is some overlap in the reservoirs identified by these markers.

Taken together, these studies suggest an association between CD32a expression and active HIV transcription in tissues. In contrast, in peripheral blood we found no enrichment for cell-associated HIV RNA in CD32^+^CD4^+^ cells, and significantly reduced HIV RNA/DNA ratios were measured in these cells compared to CD32^−^CD4^+^ cells (106), indicating that HIV is transcriptionally silent in most of the infected peripheral blood CD32^+^ cells and suggesting that CD32a indeed marks latently infected cells. These seemingly discrepant results might suggest differences in the underlying mechanisms of persistence of HIV-infected CD32^+^ cells between blood and tissues. Residual HIV replication in tissue compartments can be quite prominent due to poor antiretroviral drug penetration (80–82, 115, 116) and CD32a expression in CD4^+^ T cells is induced upon *in vitro* HIV infection (29, 99, 100, 102). Therefore, in tissues, CD32a could mark cells that were recently infected despite ART. Alternatively, CD32a expression may be up-regulated upon re-activation of HIV transcription in long-lived latently infected cells in tissues, although the reverse association between HIV transcription and CD32a expression in peripheral blood makes this possibility less likely. To study the association between CD32a expression and HIV transcription in tissues, it will also be important to determine the relative contribution of CD32b^+^ T-B cell doublets vs. *bona fide* CD32a^+^CD4^+^ T cells to the total pool of tissue CD32^+^ T cells, and to which extent these two cell types are associated with active HIV transcription vs. viral latency. Interestingly, Thornhill et al. observed a positive correlation between the frequency of Tfh cells in CD32^+^ T-B cell doublets and total HIV DNA in the rectum (but not terminal ileum or PBMC) (107), suggesting that these doublets might be associated with a higher level of HIV infection in some sites. As B cells are capable of transmission of infectious HIV to T cells via CD21-complement interactions or DC-SIGN-mediated virion capture (117, 118), some of the observed T-B cell doublets can even be involved in residual B-to-T-cell HIV trans-infection in tissues.

Despite the considerable interest triggered by the Descours study (99), many questions with regard to the CD32a-HIV link are still open. Mechanisms of CD32a expression in HIV-infected cells remain to be investigated, as well as the role of CD32a in HIV reservoir formation and/or persistence. Notably, the contribution of CD32^+^ CD4^+^ T cells to the HIV reservoirs seems to be highly variable from one HIV-infected individual to the other, both in peripheral blood and in tissues (94, 99, 100, 106). This may restrict the efficiency of CD32a therapeutic targeting for HIV reservoir eradication in some individuals. In general, targeting of CD32a alone is not a feasible option because this would deplete the majority of an individual's myeloid immune cells. However, advanced targeting strategies (e.g., bi- and tri-specific antibodies) can be used to guarantee high specificity of CD32^+^CD4^+^ cell targeting. Targeting downstream CD32a signaling in CD4^+^ T cells could also be a feasible alternative.

## Conclusions

The identification of a marker of latently infected cells has long been considered the “Holy Grail” of HIV cure programs (67). Several CD4^+^ T-cell subsets have been shown to harbor important HIV reservoirs, including resting memory CD4^+^ T cells. However, this picture is getting more complex every day as numerous other cell types were found to contribute to the HIV reservoirs. Activated T cells, naïve T cells, Tregs, Tfh, and their circulating counterpart, but also tissue macrophages, appear as major sites of HIV persistence.

Although much progress has been made in recent years in identifying HIV reservoir markers, no single cell surface molecule is currently able to mark all HIV-infected cells. However, some of the putative markers, including CD32a, CD30, and ICs, will likely play an important role in future studies aimed at understanding and eradicating the HIV reservoirs. Although a significant overlap was found in the expression of different HIV reservoir markers, targeting several markers at the same time in a “multicolor” approach may significantly increase the proportion of the reservoir that is eradicated.

## Author Contributions

AP, GD, and BB wrote the manuscript. AP made the figure. All authors approved the final manuscript.

### Conflict of Interest

The authors declare that the research was conducted in the absence of any commercial or financial relationships that could be construed as a potential conflict of interest.
